# Malaria Diagnosis Using a Mobile Phone Polarized Microscope

**DOI:** 10.1038/srep13368

**Published:** 2015-08-25

**Authors:** Casey W. Pirnstill, Gerard L. Coté

**Affiliations:** 1 Department of Biomedical Engineering, Texas A&M University, College Station, TX 77843; 2 Center for Remote Health Technologies and Systems, Texas Engineering Experiment Station, College Station, TX 77843.

## Abstract

Malaria remains a major global health burden, and new methods for low-cost, high-sensitivity, diagnosis are essential, particularly in remote areas with low-resource around the world. In this paper, a cost effective, optical cell-phone based transmission polarized light microscope system is presented for imaging the malaria pigment known as hemozoin. It can be difficult to determine the presence of the pigment from background and other artifacts, even for skilled microscopy technicians. The pigment is much easier to observe using polarized light microscopy. However, implementation of polarized light microscopy lacks widespread adoption because the existing commercial devices have complicated designs, require sophisticated maintenance, tend to be bulky, can be expensive, and would require re-training for existing microscopy technicians. To this end, a high fidelity and high optical resolution cell-phone based polarized light microscopy system is presented which is comparable to larger bench-top polarized microscopy systems but at much lower cost and complexity. The detection of malaria in fixed and stained blood smears is presented using both, a conventional polarized microscope and our cell-phone based system. The cell-phone based polarimetric microscopy design shows the potential to have both the resolution and specificity to detect malaria in a low-cost, easy-to-use, modular platform.

Malaria is a life-threatening disease caused by parasites that most often infect a subject via transmission from a mosquito bite. Following infection, the parasite begins invading the host’s red blood and liver cells, modifying the biochemistry and structural properties of the cells. According to the World Health Organization (WHO), an estimated 584,000 deaths were caused by malaria in 2013 with an estimated 198 million new cases in the span of time^1^. Worldwide, the mortality rates associated with the disease have fallen; however, a significant portion of deaths still primarily affect African children^1^.

**Traditional Microscopy as the Gold Standard. ** The recommended gold standard and primary method for evaluating blood samples for malaria detection utilized around the world is the observation of Giemsa-stained thick and thin blood smears via brightfield microscopy. This technique offers the ability to detect parasitemia associated with 5–10 parasites in 1 *μ*l of blood^2^,^3^,^4^. Thick blood smears most commonly provide a positive or negative screening test to determine if the parasite is present in a blood smear, while thin smears are most commonly used to determine the species of malaria infection. Thick smears result in lysed red blood cells (RBCs), consist of larger volumes, and have greater parasite density as compared to thin smears. For thin smears, white light microscopic imaging with a higher magnification and resolution is utilized to identify the species present and evaluate parasite morphology^5^. However, conventional white light microscopy often requires a clinical laboratory structure in addition to trained microscopy technicians, which are both rare in regions where malaria is most prevalent^5^,^6^,^7^,^8^,^9^,^10^,^11^,^12^,^13^,^14^,^15^. Furthermore, current microscopy techniques results in a subjective measure, reported to vary significantly, based on the training and equipment utilized by the expert microscopy technician^16^,^17^. Thus, the need for devices capable of malaria diagnosis in remote areas has led to the design and implementation of mobile health (mHealth) based portable systems^15^.

**Rapid Diagnostic Tests for Field Use. ** Rapid diagnostic tests (RDTs) have become widely used throughout the world and offer a cheaper and less time consuming alternative for diagnosis of malaria using a finger-prick of blood^6^,^17^,^18^,^19^,^20^. However, RDTs currently employed for malaria detection consist of an antigen-based detection scheme. Thus, RDT performance has been reported to degrade in tropical areas where the disease is most prevalent due to the sensing chemistry^3^,^21^. Additionally, existing RDTs detection limits tend to be much higher than the current gold standard tool for detection of early-stage infections. The current sensitivity threshold for RDT tests is greater than 100 parasites/*μ*l of blood. Further, these devices do not provide quantitative parasitemia results and suffer from reported inconsistent performance, specifically in diagnosing strain specific malaria infections^2^,^6^,^22^. Additionally, RDTs are ineffective at diagnosing low parasite densities^20^. This lack of quantification of the parasitemia level does not allow identification of patients requiring urgent treatment^20^,^23^. A recent study reported that a substantial misdiagnosis rate for malaria currently exists in parts of Asia using RDTs^24^,^25^. Misdiagnosis then leads to substantial mistreatment and over diagnosis of malaria throughout central and south Asia, often resulting in wasted drugs and potential immunity build up in these populations to the anti-malaria drugs^24^,^25^. Accurate diagnosis has become more important as drug resistant malaria continues to spread, causing medications to become more costly^3^.

**Other Microscopies Using Contrast Enhancement from Malaria Hemozoin Crystals. ** Other traditional microscopy techniques used for malaria diagnosis have included cross-polarization microscopy and dark-field microscopy^3^,^5^,^6^,^7^. Both techniques utilize imaging contrast enhancement from hemozoin to improve diagnostic capability for the presence of malaria in blood smear samples^5^,^6^,^7^,^20^,^26^. Hemozoin crystals occur in varying amounts within malaria infected blood smear samples. The crystals are created as the byproduct of hemoglobin metabolism by the malaria parasite in an infected host and are optically birefringent, meaning they result in rotation of the plane of polarized light that pass through the crystals based on the anisotropies^2^,^7^,^23^,^26^,^27^,^28^,^29^,^30^. Since hemozoin is a birefringent compound, it is much easier to observe under polarized light microscopy as compared to traditional white light microscopy. Typically the size of individual hemozoin crystal rods vary between approximately 300 nm to 1 micron in length with widths between 10–20 nm^2^. However, during the process of producing these crystals they often exist in larger clusters of individual hemozoin crystals that can vary in size^2^. Specifically, studies using polarized microscopy that can illuminate these highly birefringent hemozoin crystals have shown that this technique may even be better than conventional staining, particularly for the less severe cases of malaria infection^7^,^27^. Maude *et al.* demonstrated that when examining histologic specimens, polarized microscopy sensitivity was approximately double that of conventional light microscopy for the detection of the malaria parasite^7^,^26^. Specifically, the minimum concentration of hemozoin that can be measured with polarized light is 15 picograms^2^. Further, this hemozoin level is correlated to the lower limit of detection currently reported for parasitemia, namely 30 parasites/μl of blood^2^.

The conventional staining approach is also potentially inferior because it can include many false positive indications for the presence of malaria, specifically in diagnosis of tissue samples in placental malaria diagnosis^7^,^26^. However, similar to traditional white light microscopy, polarized microscopy is rarely implemented for field-based diagnosis or even in the clinic because the technique requires costly and complex microscope configurations, sophisticated maintenance, and the microscope systems tending to be bulky in size^1^,^2^,^6^,^7^,^27^,^31^,^32^,^33^,^34^. Thus, further research in the design, development, and testing of low-cost, easy to use, polarized microscopic imaging systems as an alternative approach to current microscopy in malaria diagnosis is needed. The polarized light cell-phone design described in this report intends to overcome these drawbacks and assist medical professionals in the clinic and in low-resource settings to improve correct diagnosis of malaria with enhanced detection via a field-based modular polarized microscope.

**Use of Mobile Phone Technology for Medical Diagnosis in Remote Areas. ** In general, mobile phones offer an ideal platform for creating a field-based, modular polarized microscope. Currently, over 6 billion cell-phone subscriptions exist worldwide (accounting for approximately 75% of the world having access to mobile phone networks)^35^,^36^, with the vast majority of these users (~5 billion) located in developing countries. Utilizing the existing mobile infrastructure allows for significant reduction in cost and size of mobile based designs as compared to traditional microscopes^35^,^36^. Additionally, the number of active mobile phone subscriptions is continually increasing, particularly in low-resource settings, and is expected to surpass the world population by the end of 2014^37^. Due to the large volume of wireless communication users, mobile phones continually remain at relatively low-costs even with constant advancements in hardware and software specifications on new models. Because of increased access to these mobile networks and the fact that many mobile phones currently available are equipped with advanced camera features and other technologies, they have become an ideal platform for many advanced imaging and sensing mHealth applications resulting in several portable field ready point-of-care (POC) devices^5^,^35^,^38^,^39^,^40^,^41^,^42^,^43^,^44^. These mobile point-of-care (POC) platforms offer great opportunities for improved healthcare throughout the world by offering high quality alternatives to existing imaging modalities that are low-cost, portable, and energy efficient. This is particularly important for the clinic and field in remote areas and in low resource settings, where the medical infrastructure is often times limited or non-existent.

More specifically, a significant number of studies have focused on introducing new cell-phone based systems that provide low-cost alternatives to conventional microscopy techniques for mHealth applications including malaria detection^5^,^40^,^41^,^42^,^43^,^44^. Mobile phone based microscopy approaches can be broken down into three specific design areas including: lensless approaches^40^,^42^, on-lens approaches^41^, and attachment based approaches^5^,^43^,^44^. Research in each of these design categories has produced promising scientific approaches towards cell-phone microscopy designs capable of significantly affecting healthcare standards in developing countries, particularly in the area of single cell resolution for disease diagnosis. Several cell-phone based microscopy designs for malaria diagnostic applications have been reported.

Holography is used in the lensless design to enable resolution comparable to traditional brightfield microscopic techniques (~40x magnification, NA = 0.65 objective) utilizing post-processing techniques of images collected by the camera on a mobile device. Using a lensless design approach can allow for more compact designs and eliminates the need for optical alignment^40^,^42^. Additionally, this technique allows for decoupling of the relationship between field-of-view (FOV) and resolution, thus allowing for significant improvements in large FOV imaging without sacrificing system resolution as compared to traditional microscopy techniques^42^,^45^,^46^,^47^,^48^,^49^,^50^. This design approach has resulted in systems used for detecting the presence of malaria parasites on standard blood smears^35^,^38^,^43^,^45^,^46^,^51^,^52^,^53^,^54^,^55^,^56^. There are two main limitations that restrict this approach for certain applications. **(1)** The cell-phone microscopy systems used in this configuration must contain enough processing power to reconstruct the resultant images from holograms or process the images remotely on a server. **(2)** The samples to be imaged must be placed relatively close to the camera^42^,^45^,^46^,^47^,^48^,^49^,^50^.

The second approach uses on-lens device design configurations typically employing a refractive element directly attached to the cell-phone camera at the focus, or a ball lens mounted in front of the camera lens^41^,^44^. This approach allows for a low-cost alternative and produces comparable resolution to other reported cell-phone based microscope systems. The ball lens creates a spherical focal plane; thus, the technique only allows for a small FOV of a captured image to be in focus. The out of focus areas in the FOV then need to be adjusted using image processing correction techniques^41^,^44^.

The third approach, incorporates the majority of reported cell-phone based microscopy designs, uses an attachment method. In this approach, additional hardware is required for microscopic imaging^5^,^38^,^57^. The hardware attachment often consists of a clip-on attachment used to mount hardware such as a commercial objective or low-cost singlet lenses to a cell-phone^44^. The limitations of this approach are that a separate attachment is required for each individual cell-phone model and complex optical elements may be required, thus potentially limiting the application of such a design in developing countries.

In this report, we describe the design of a low-cost, lightweight, high quality mobile-optical-polarization imaging device (MOPID) with similar resolution and field-of-view (FOV) compared to larger bench-top polarized microscopy systems for POC diagnosis of malaria. The device characterization and preliminary results presented here illustrate these key advantages of a MOPID for malaria diagnosis over established detection modalities. In addition to providing advantages such as improved contrast of images containing malaria-infected blood, this technique also allows for a reduction in the time a skilled professional would need to examine a test slide for determining the presence of an infection.

## Results and Discussion

**Figure 1:**
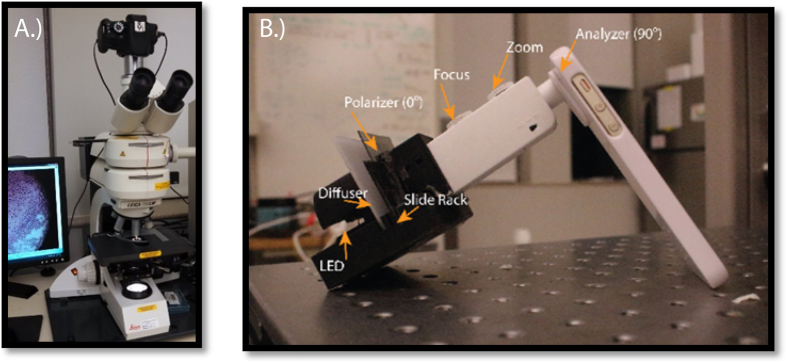
(**A**) Leica DMLM polarized white light microscope used as reference for comparison in this study; and (**B**) a microscope lens combination implemented into a 3D-printed fitting to allow similar function to a traditional polarized laboratory microscope. The MOPID system was configured in transmission mode with a magnification designed for 40X when using a mobile phone camera. An iPhone 5s was used with polarizer sheets added and a 3D-printed fitting to hold the light source, diffuser, sample slide, and microscope attachment.

A Leica microscope was used as the gold standard and compared to the designed brightfield and polarized brightfield optical instruments that were attached to an iPhone 5s cell-phone as described in the experimental methods section below and as shown in Fig. 1.

### Resolution and Field of View Performance Testing

**Figure 2:**
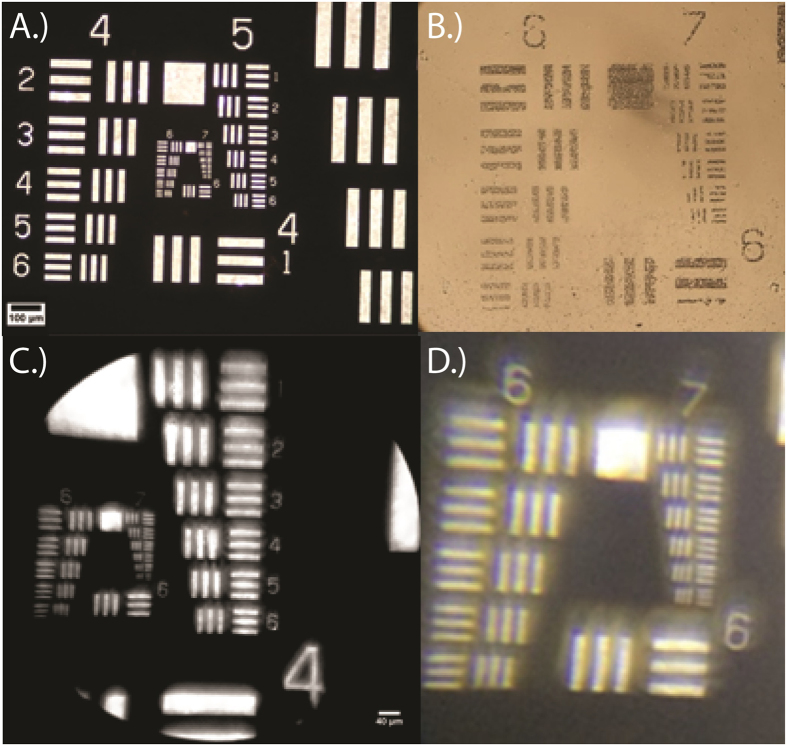
USAF resolution target images were utilized to determine FOV, resolution, and other optical system parameters with a reference Leica microscope that included (**A)** a 20x magnification; and (**B)** a 40x magnification. The USAF target images on the bottom were acquired using the polarized mobile platform with (**C)** full zoom with microscope attachment for cell-phone on iPhone 5s and (**D**) Digitally zoomed image taken from the cell-phone image shown (**C**).

Individual United States Air-Force (USAF) resolution target images (Edmund Optics, Barrington, NJ) are shown in Fig. 2, using both the reference microscope and constructed MOPID device, each equipped for transmission mode imaging. The approximate magnification achievable by the MOPID at the camera face was determined to range from 40X to 100X depending on the sample, illumination settings, and the FOV for the individual images acquired with the mobile phone camera. The overall full-width half maximum (FWHM) was calculated by averaging over the total number of FWHM values individually determined for the sample based on different Group and Element measurements for each image.

**Figure 3:**
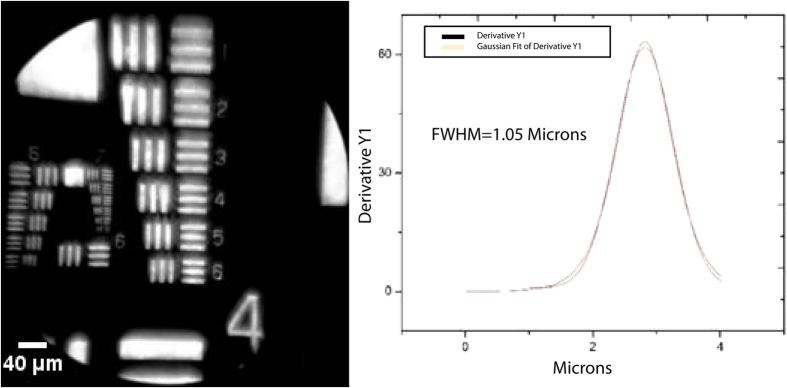
IPhone 5 s **(left)** configuration USAF target image and (**right**) the calculated derivative for a line spread across Group 7 Element 6. FOV was 0.78 mm × 0.79 mm.

Using Group 7 Element 6, the smallest group on the USAF resolution target, without using the devices digital zoom function, the FOV for each configuration was determined. From the acquired images, shown in Fig. 2B–D and 3, the FOVs were calculated to be 0.42 mm × 0.24 mm for the reference microscope with a 40x objective and 0.78 mm × 0.79 mm for the MOPID configuration shown in Fig. 1B that was attached to an iPhone 5s cellular phone. Next, calculations for spatial resolution were determined using the FWHM measurement of a fit function for the derivative of the boundary line intensity value between light and dark regions on the USAF target. Calculating the total effective system magnification occurred after careful interpretation of acquired USAF target image features with known distance measurements. This magnification varied from the approximate magnification value at the camera face because of digital enlargement capabilities of the acquired image via the camera software settings on the iPhone 5s before capturing the photograph.

Using the highest resolvable Group (Group 7 for iPhone 5s configuration) from the USAF resolution target, the systems spatial (lateral) resolution was determined to be ~1.05 μm and the reference Leica determined to have a resolution of ~0.47 μm. The measured resolution is a factor of 2.6 larger than the nominal Rayleigh resolution limit of 0.4 μm for the portable system. An expected increase in measured resolution as compared to nominal Rayleigh resolution limit occurs because the optical components utilized in the construction of the MOPID consist of low-cost plastic lenses. Poor lens selection results in improper correction for field of curvature and additional aberrations that are present in the system, resulting in reduced resolution away from the field radius of best focus. In addition to the plastic microscope lens components, the mobile phone camera lens assembly also contributes to reduced system resolution observed, resulting in non-diffraction limited performance. However, as previously reported with many brightfield cell-phone microscope designs^5^,^41^,^44^, the system limitations did not hinder the mobile phone camera from being able to capture high-definition (HD) images of malaria infected blood smear samples and additional non-malaria samples allowing for useful diagnosis and comparison between reference images utilizing a commercially available laboratory microscope.

In this study, determination of FOV and resolution for the acquired images was important for comparing the acquired images using the MOPID device with the reference images and to verify a minimum metric can be achieved when employing traditional parameters such as shape of parasites present or ratio of malaria infections to RBCs present within a sample for determining if an infection is present. These parameters are additionally useful in determining malaria strain type, parasitemia level, and if an infection is present.

While many parameters can be used to compare the two images, the main constraints in the design described are that the overall system resolution needs to be adequate to measure within the individual red blood cells (RBCs) (<5 microns) in order to see some instances of the presence of hemozoin within an infected blood smear sample. Thus, better resolution of the system provides an increased likelihood to observe the presence of individual malaria infected birefringent clusters within a given sample volume. The FOV measurement is important not only for comparative purposes with the gold standard images but also for determining the number of total fields that would be required to image in order to provide relevant diagnostic capability with a limit of detection less than 30 parasites/μl. For example, current microscopy experts determine the number of malaria parasites compared to the number of red blood cells in up to 100 field-of-views to provide a final determination as to whether the sample is considered infected or uninfected with malaria. In the proposed setup, a larger imaging FOV, without sacrificing the ability to diagnose individual malaria parasites, allows for less individual images needed to properly diagnose parasitemia levels in the sample.

### Non-Malaria Polarized Light Comparative Sample Images

**Figure 4:**
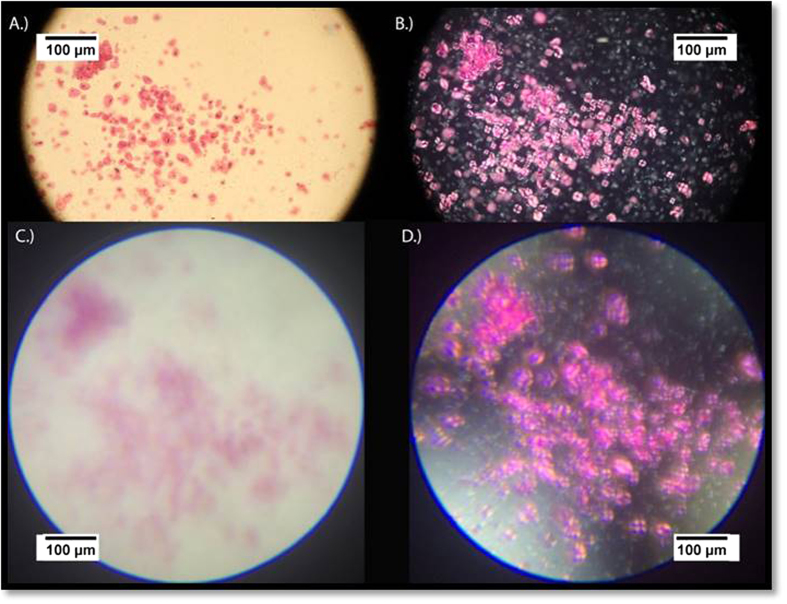
Images of a microscope slide coated with wheat starch acquired using a Leica microscope with a 40Xobjective with (**A)** no polarizers present in the imaging plane and (**B)** with a polarizer and analyzer crossed at 90 degrees in the imaging plane. For comparison, the reference an iPhone 5s utilized to acquire images of the same location on the wheat starch slide were acquired with (**C)** no polarizers present; and (**D.)** with polarizer and analyzer crossed at 90 degrees. In both setups, the polarized images illustrate the presence of a Maltese cross for each starch molecule.

Prior to characterizing the MOPID towards the clinically relevant malaria application, non-polarized and cross-polarized images were evaluated from the same area of a slide containing wheat starch. The resolution of the images acquired with each system were compared in addition to evaluating if the classic Maltese cross could be depicted from the polarization changes as light transmits through the wheat starch molecules. Indeed, in Fig. 4B,C the starch molecules exhibit a Maltese cross configuration. Additionally, using a 40X objective with an NA of 0.65 on the Leica DMLM microscope comparable FOV and resolution were achieved for the MOPID images acquired over the same area.

The image acquisition conditions for the cell-phone images and the Leica images are listed in Tables 1 and 2. For the iPhone setup used to obtain the wheat starch images, integration times of 1/255 seconds and 1/30 seconds were used. These were based on the mobile phones auto integration/exposure setting with ISO speeds of 32 and 80 for the non-polarized 4C and polarized 4D images, respectively. The need for longer integration time in image 4D is based on the low light present in the cross-polarized configuration versus the normal light configuration. Additionally, the corrected focal length for each image was 30 mm and the magnification was set to 0.25.

### Brightfield and Polarized Imaging of Malaria

**Figure 5:**
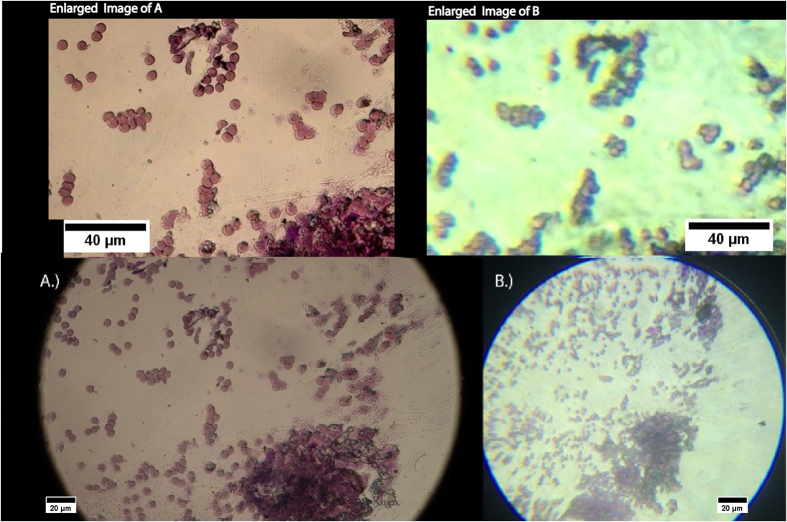
Images acquired of mouse malaria strain blood smear without polarized light using (**A**) a Leica microscope with a 40X magnification objective and (**B**) the same area of the slide imaged utilizing the iPhone 5s mobile phone based design. Above each of the respective images is a zoomed in image of the same region for each photo to better illustrate the comparable resolution of the two microscopes.

Comparative brightfield images acquired to analyze a specific zoomed in section on the infected thin blood smear with the reference microscope in non-polarized transmission mode and the MOPID in non-polarized mode are shown in Fig. 5. To compare the respective images acquired from each system, to further illustrate the minimal resolvability of single cell characteristics utilizing the MOPID, each of the images acquired in Fig. 5A–B were enlarged and cropped, as shown with the respective images above Fig. 5A–B. These cropped images represent a smaller region within the total image for evaluating individual RBC resolution. Figure 5 illustrates that the low-cost high resolution MOPID is capable of achieving <2 μm resolution. The Leica reference design resolution was calculated to be ~0.47 μm.

Images shown in Fig. 5 (Enlarged Image A and B) illustrate that the MOPID has the high resolution of the Leica microscope reference and it is capable of resolving single RBC boundaries in many cases where overlap of the individual RBCs is not extensive. Although, this could potentially be a problem in traditional microscopic histological examination of the malaria-infected blood smears, it is not an issue in the proposed polarized light cell-phone based setup because of the fact that enhanced contrast is achieved when examining the cross-polarized images in the presence of birefringent variation caused by the hemozoin in the sample.

**Figure 6:**
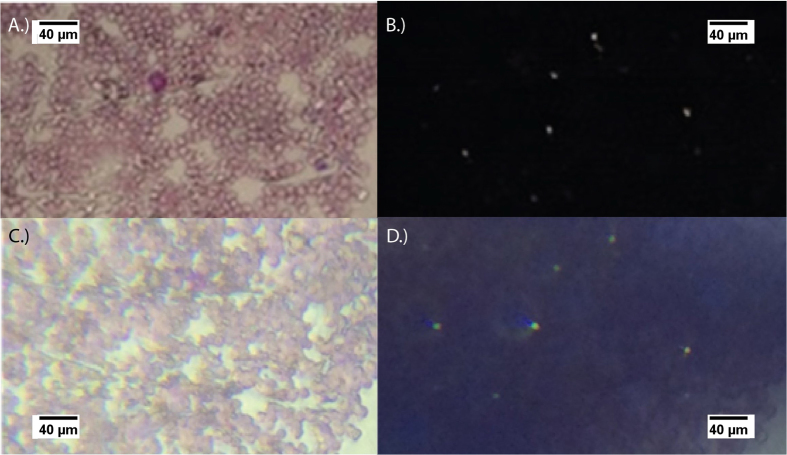
Images of a Giemsa stained mouse blood smear using a Leica microscope with a 40X objective and (**A)** no-polarizers present in the image plane and (**B)** with a polarizer and analyzer crossed at 90 degrees in the image plane. The iPhone 5s utilized to acquire images of the same location on the prepared microscope slide with (**C)** the mobile phone polarized microscope system having no polarizers in the system and (**D)** the same system including a polarizer and analyzer crossed at 90 degrees in the image plane. In both crossed images, the birefringent hemozoin correspond well.

To show the contrast polarized light microscopy provides from thin smears of *Plasmodium chabaudi* malaria-infected blood samples, the images in Fig. 6 are presented. Specifically in Fig. 6A,B, brightfield non-polarized and polarized thin Giemsa-stained blood smear samples of malaria-infected RBCs at 40X magnification images were obtained via a digital SLR camera mounted onto a Leica DMLM polarized microscope. As indicated by the presence of birefringent changes in the polarized reference image, Fig. 6B, the sample had positive infected areas with the malaria-parasite. The presence of hemozoin particles in the sample cause the polarized transmitted illumination light to vary in intensity and wavelength due to variation in the light as it transmits through the birefringent hemozoin particles. The result of this change is represented by seven bright white dots that appear in the cross-polarized reference image. It should be noted that it is very challenging to detect these hemozoin particles in the original non-polarized reference imaging system without being a highly trained technician. This confirms previous reports, by Maude *et al.* and others, that the use of polarized microscopy in observing the presence of malaria-infected RBCs has shown to improve diagnostic capability up to two fold in some instances^7^,^26^. Following the acquisition of the two reference images, two additional images, a non-polarized and polarized image, were capture from the same malaria-infected sample and sample region of the blood smear with the MOPID and are shown in Fig. 6C,D. It is clear from the non-polarized images in Figs 5 and 6A,C that the mobile platform has a reduced system resolution as compared to the reference microscope in polarized mode. However, in examining the polarized images from both systems it is clear that the presence of birefringence appears at the same spots within the sample. This indicates that the results obtained with the MOPID are capable of determining the presence of malaria with lower system resolution, and with less user expertise than traditional microscopy requires.

The main advantages of the system described in this report are the reduction in cost and complexity associated with conducting polarized microscopy for malaria detection on a mobile platform as well as a reduction in the need for a trained microscopy expert to diagnose the presence of malaria in a blood smear sample in the field. This significantly increases the potential application for the approach in addition to increasing the likelihood of adoption of the technique in developing countries where cost, complexity and lack of expertly trained technicians can often prohibit the use of a polarized microscopy technique or even traditional laboratory microscopy as the standard of diagnosis.

The image acquisition settings for the images acquired with the cell-phone and Leica microscopes are listed in Tables 1 and 2. The iPhone blood smear images, Figs 5B and 6C–D, had integration times of 1/1580 seconds, 1/1642 seconds, and 1/30 seconds based on the mobile phones auto integration/exposure setting with ISO speeds of 32, 32, and 80 for the non-polarized Figs 5B and 6C and polarized 6D images respectively. The difference in value is determined based on the low light setting in the cross polarized configuration versus the normal light configuration. Additionally, the corrected focal length for each image was 42 mm, 150 mm, and 150 mm with the magnification set to 0.17, 0.25, 0.25. Additionally, Fig. 5B had a digital zoom ratio of 1.4 while Fig. 6C,D having a 5x digital zoom ratio.

An independent validation of the presumed malaria infected birefringent areas for the sample area provided in the report was performed utilizing the reference microscope configured in a traditional white light microscopy orientation. With this setup, polarized and non-polarized images were evaluated and compared within the region suspected of infection. Each birefringent area was observed closely to determine if the birefringent area occurred within a cell body that appeared to be infected based on traditional metrics such as shape and color properties. Further, the birefringent area was evaluated to determine if the change occurred in plane with the cellular components of the sample. Often, contaminants that may be present from the preparation process will exist out of plane with the cellular components of the sample. From reported literature, dust or dirt is the primary component that can often contaminate a sample during the preparation procedure. If present, the dust or dirt can often appear very similar to changes observed from the presence of hemozoin in the sample. Although, the dust also generates changes in the state of polarization, the changes primarily occur out of plane from the sample RBCs^2^,^7^. To determine if the birefringent area was in fact generated from the presence of hemozoin the following criteria were utilized in each polarized image: **(1)** a comparison of the non-polarized and polarized image verifying the birefringent area occurs in the same region of a cell in the non-polarized image. **(2)** determination if the birefringent area occurred in the same image plane or close to the image plane of the cellular components and **(3)** the coloring and shape of the cellular components in the proximity of the birefringent area were evaluated to determine if they are consistent with reported changes due to the presence of a malaria infection.

## Materials and Methods

### Polarized Light Systems

A commercial non-inverted polarized Leica DMLM microscope (Leica, Germany) was used as the gold standard for imaging (Fig. 1A). The system was equipped with a 20X or 40X objective coupled to a commercial Cannon Rebel T3i digital SLR camera (Cannon, Melville, NY), as shown in Fig. 1A. For the MOPID system the optical setup and components (Fig. 1B) consisted of a commercial cellular phone, customized attachable mount, two polarizer sheets (that could be removed for non-polarized imaging), low-power high efficiency white light emitting diodes (LEDs), and a plastic lens assembly configuration allowing for appropriate magnification, resolution, and FOV for diagnosing the presence of the malaria parasite.

The Apple (iPhone 5s) camera-enabled cell-phone was used as the base unit. The iPhone 5s employs an 8-Megapixel iSight camera with a CMOS back-illuminated sensor (BSI). The camera has a physical sensor size of 1/3” or 8.47 mm, with pixel dimensions of 3264 × 2448 composed of 1.5 μm pixels. The camera also features an autofocusing lens and consists of a 5-element plastic lens combination on with an aperture size of f/2.2 values shown in Table 2.

The designed system utilizes a low-cost plastic lens assembly adjustable to achieve different magnification, resolution, and FOV parameters depending on the desired system and sample specifications. An inexpensive microscope plastic lens assembly (GadgetZone, U.S.), with adjustable focus and zoom between 160X-200X, was attached at the focal point of the camera on the back of the iPhone. The microscope assembly is fitted to the iPhone 5s using a modified plastic phone case (CARSON Optical®, Ronkonkoma, NY) with an open port available to attach the microscope lens assembly positioned at the camera face. To achieve the range of magnification specified for the product, digital zoom implementation on the mobile phone was used in combination with the optical zoom of the constructed MOPID.

Following the adjustable multiple lens microscope assembly in the MOPID design, a snap on 3D-printed cartridge had individual compartments that allowed for polarized microscopy. The cell-phone 3D-printed cartridge attachment consisted of PLA plastic using a fused deposition modeling 3D-printer (MakerBot Industries, New York NY). The attachment included slotted areas to insert the sample microscope slide, a rotatable polarizer sheet, titanium dioxide chip diffuser plate (RTP Company, Winona, MN), and white LEDs (COAST, Portland, OR) to illuminate the sample. The ability to rotate the analyzer, varying the degree of polarization, allows for both conventional histology and crossed polarized light imaging for birefringence measurements from the hemozoin crystal.

The portable commercial microscope lens assembly consisted of two separate plastic lens modules and had a numerical aperture (NA) value of 0.65. The MOPID design incorporated white light LEDs placed at a distance from the sample, allowing for even field illumination. The individual LEDs were chosen because of their low-cost, low power, durability, and long lifetime; all characteristics ideally suited for use in low-resource settings^5^,^13^,^58^,^59^. In the designed system, the white light LED total power was 66 lumens after the light passes through a TiO_2_ diffuser plate. The diffuser plate is utilized to allow for homogenous illumination across the sample and was followed by a polarizer sheet generating linearly polarized light prior to transmission through the sample. The opening in Fig. 1B, labeled slide rack, is the location where the blood smear or histological slide is inserted into the optical train. This slit is located such that the sample is optimally positioned at approximately one focal length of the imaging system. Utilizing a multi-position insert the slide can be manually moved past the camera from left to right in incremental steps. The final component prior to the mobile phone surface in the optical path is a second polarizer, an analyzer, capable of being oriented either at 45 or 90 degrees with respect to the initial polarizer orientation.

### Blood Smear Sample Preparation

Sample blood smear slides were prepared by collecting single drops of blood obtained from BALB T cell receptor (TCR) transgenic mice infected with the *Plasmodium chabaudi* malaria parasite. The mice were infected and the blood smear samples collected on day 8 of the infection. To prepare the smear, a drop of blood was placed onto a glass microscope slide and then smeared across the slide using the edge of a second glass slide. Prior to acquiring images from the blood sample, the blood smear undergo fixation with methanol and Giemsa-staining. If the malaria parasite is present once the sample is Giemsa-stained, the virus becomes visible via microscopy to the trained eye as a purple ring that typically forms in the RBCs when present. No live animal studies were performed in this research. All methods were approved by Texas A&M University System and carried out in accordance with approved University guidelines. Specifically, the Malaria slides were obtained from another study, which the methods were carried out in “accordance” with their approved guidelines. All experimental protocols were approved by UTMB’s internal review board for the animal preparation as part of another study. The slides were then acquired per a loan agreement.

### Method for Polarized Light Microscopy Imaging

Polarized images acquired with the MOPID and Leica microscope setups described above allowed for comparison between the two systems. The two systems were comparatively evaluated based on the following criteria: 1) Resolution, 2) Field of view (FOV), 3) Magnification, and 4) Illumination quality.

The depth of focus, illumination beam quality, and divergence angle on the FOV, was calculated for each system. Additionally, the predicted system magnification was determined for the MOPID combination. Similarly, the NA of the system was calculated and compared to the reported data. Using this number, the diffraction limited resolution was calculated for the MOPID and reference design setup described above. Utilizing each hardware configuration and the different lens combinations, USAF resolution target images were acquired in addition to images of prepared samples on microscope slides. The USAF target images acquired from the designed MOPIDs were analyzed using ImageJ to determine field of view based on the known size of the rectangular elements of each group on the USAF target. Using the recorded camera settings for focal length, pixel spacing, number of pixels, and f-number, the theoretical limits of the imaging systems were calculated. Using these calculated values and the USAF target images, the overall system magnification, FOV, and resolution were calculated for each configuration. As described above determing the FOV and resolution of each image is important with the MOPID system because these two measurements are the primary metrics utilized to compare the MOPID design against the gold standard Leica microscope.

### Image Analysis

To determine the resolution, FOV, and other optical parameters of each image acquired for the two systems, both the ImageJ Fiji plugin and Origins Pro (version 8) statistical software were utilized. Initial images were acquired from the two respective systems and each opened in ImageJ for processing and analysis of the individual images. To convert the measurement area of each photograph into micrometer units for FOV calculations and resolution measurements, the Set Scale command in ImageJ was utilized. For each image captured, a straight line was manually placed over the image corresponding to a known distance in the microscope stage image. Using the constructed line and known distance, a unit of measurement entered into the Set Scale command dialog for conversion allowed for transformation of the pixels to distance. ImageJ was used to auto populate the distance in the pixels field based on the length of the line drawn^26^. Following the conversion of length to calculate the resolution, the following sequence of analysis occurred. Starting with a known Group and Element number on each USAF target, a constructed line was placed vertically from the black background across the white bar edge of a known length. Using the plot values command in the analyzed pull-down, the line x and y values were listed. Using the values, a plot was generated in Origins from analyzing the shape of the output profile at the edge between the dark and light boundary. Additionally, manual insertion of a scale bar on each image using the ImageJ software occurred followed by saving the images as a jpeg extension and utilizing them in this report.

In Origins Pro, after plotting the graph of the boundary, the curve was fitted with a sigmoid function from the analysis pull-down using a Boltzmann function relationship. Using the generated sigmoid fit function, a new plot resulted consisting of the derivative of the sigmoid function. From the derivative curve plot, a non-linear fit function with a Gaussian fit was used to generate the derivative curve. From this fit, a FWHM of the spatial resolution was generated for each imaging system. For each USAF target image, multiple Groups and Elements combinations were averaged over all FWHM values to develop an approximate resolution value.

## Conclusions

In summary, a polarized microscope platform design using a cell-phone is shown for the first time to be capable of detecting birefringence in histological specimens infected with the malaria parasite. The MOPID is a simple, low-cost, design capable of easily being adapted to multiple mobile device platforms. Device resolution was determined to be sufficient for observing birefringence from hemozoin crystals. Based on USAF resolution target images, the designed system using an iPhone 5s was determined to have a resolution of 1.05 μm, a system magnification of ~50X, and a FOV of 0.78 mm x 0.79 mm. While the non-polarized RBC images are difficult to diagnose, the cross-polarized images clearly indicated the presence of hemozoin in the sample with resolution comparable to images from a reference Leica DMLM polarized microscope.

Resolution and FOV measurements for the proposed system were important in determining the number of fields that would be required to determine accurate parasitemia measurements within an infected sample. Further, FOV and resolution measurements become more significant when polarization measurements are used as a contrast enhancement method to calculate automated detection of malaria presence, parasitemia, and strain information for a given sample including being able to determine the location of the hemozoin birefringence relative to the red blood cells. When exploring these metrics, polarization in combination with object shape and total cell number become important in providing the desired calculation and thus require a resolution to at least determine red blood cell size.

The focus of this research moving forward is to use human strains of malaria and to construct a more compact device, improving durability, usability, and decrease the cost for *in vivo* field-testing in Rwanda, Africa. Specifically, we envision the final product being available for less than $1.00 per test result. The MOPID described here cost around $7.00 since the unit consisted of a commercial microscope lens attachment for the design. This total cost estimate does not include the cost of the mobile phone attached to the MOPID device. The envisioned final product design can be made available to multiple commercially available phones to provide an attachment to users existing phone platforms.

## Additional Information

**How to cite this article**: Pirnstill, C. W. and Coté, G. L. Malaria Diagnosis Using a Mobile Phone Polarized Microscope. *Sci. Rep.* **5**, 13368; doi: 10.1038/srep13368 (2015).

## Figures and Tables

**Table 1:** Figures 4–6 image settings.

**Figures**	**4A**	**4B**	**4C**	**4D**
Integration Time (sec)	1/10	1/100	1/255	1/30
ISO Speed Ratings	100	800	32	80
Focal Length 35 (mm)	−	−	30	30
Magnification	0.17	0.17	0.25	0.25
**Figures**	**5B**	**5B**		
Integration Time (sec)	1/10	1/1580		
ISO Speed Ratings	100	32		
Focal Length 35 (mm)	−	42		
Digital Zoom Ratio	1	1.4		
Magnification	0.25	0.17		
Figure 6	**6A**	**6B**	**6C**	**6D**
Integration Time (sec)	1/10	1/100	1/1642	1/30
ISO Speed Ratings	100	800	32	80
Focal Length 35 (mm)	−	−	150	150
Digital Zoom Ratio	1	1	5	5
Magnification	0.25	0.25	0.25	0.25

**Table 2:** iPhone 5 s settings.

**CMOS Sensor**	**X**
Sensor Format	(4.89 × 3.67 mm)
Optical Elements	5 Plastic
Pixel Size	1.5 μm
Focal Length	4.12 mm
Aperture	F/2.2
Image Capture Size	3264 × 2448
